# Innovative mHealth for addressing low back pain-related factors amongst lower-limb amputees: A quasi-experimental

**DOI:** 10.4102/ajod.v15i0.1890

**Published:** 2026-05-07

**Authors:** Muh Syaiful Akbar, Muhammad Syafar, Yahya Thamrin, Djohan Aras, Indar Indar, Muhammad Arsyad

**Affiliations:** 1 Doctoral Program of Public Health, Faculty of Public Health, Hasanuddin University, Makassar, Indonesia; 2 Department of Biology, Faculty of Mathematics and Natural Sciences, Makassar State University, Makassar, Indonesia; 3 Department of Health Promotion and Behavioural Science, Faculty of Public Health, Hasanuddin University, Makassar, Indonesia; 4 Department of Occupational Safety and Health, Faculty of Public Health, Hasanuddin University, Makassar, Indonesia; 5 Department of Physiotherapy, Faculty of Nursing, Hasanuddin University, Makassar, Indonesia; 6 Department of Hospital Management, Faculty of Public Health, Hasanuddin University, Makassar, Indonesia

**Keywords:** lower-limb amputation, low back pain, mHealth, quality of life, disability

## Abstract

**Background:**

Lower-limb amputees (LLAs) are highly susceptible to low back pain (LBP) because of postural instability and core muscle weakness, leading to reduced quality of life (QoL). Tailored mobile health (mHealth) interventions may offer a scalable strategy to support functional improvement.

**Objectives:**

This study aimed to evaluate the preliminary impact of mHealth applications on QoL and to identify behavioural and physical parameters observed on LLAs.

**Method:**

A quasi-experimental one-group pre-test–post-test longitudinal study was conducted with 22 unilateral LLAs (transtibial or transfemoral). Nineteen participants used a prosthesis, while three participants used a wheelchair. Participants engaged with a structured 3-month intervention delivered through an mHealth application. The programme consists of: (1) educational modules; (2) core-strengthening exercises; (3) automated reminders; and (4) hybrid delivery. Quality of Life was assessed using the WHOQOL-BREF, variables examined in the bivariate analyses and changes in outcomes were analysed using Wilcoxon signed-rank tests.

**Results:**

The Wilcoxon signed-rank tests showed significant improvements in positive attitude (*p* = 0.007) and good core muscle strength (*p* = 0.020), both with large effect sizes. Descriptive analysis indicated significant overall changes in QoL. Bivariate analyses suggested that positive attitude and good core muscle strength were associated with higher QoL.

**Conclusion:**

The use of the mHealth application was associated with improvements in QoL, and positive behavioural and physical changes among LLAs.

**Contribution:**

This research contributes to disability rehabilitation in Southeast Asia by introducing an mHealth approach that may improve functional improvement among LLAs.

## Introduction

Low back pain (LBP) is one of the leading global health problems and remains the most common cause of disability worldwide. According to the Global Burden of Disease Study, the burden of LBP has steadily increased, with substantial rises in years lived with disability and disability-adjusted life years between 1990 and 2019, highlighting its growing impact on public health and healthcare systems (Chen et al. 2022; Ferreira et al. 2023; Yang et al. 2023). The lifetime prevalence of LBP is estimated at up to 80%, and its chronic forms often restrict daily activities, reduce work productivity and impair quality of life. Moreover, LBP is frequently accompanied by psychological comorbidities such as anxiety and depression (Beyera, O’Brien & Campbell 2022; Pinto et al. 2023; Sirbu et al. 2023). Individuals with lower-limb amputation are at disproportionately high risk of developing LBP compared with the general population. This vulnerability is attributed to musculoskeletal disorders following amputation, including asymmetric gait patterns, postural instability and core muscle weakness, which are associated with functional limitations and reduced quality of life (Al-Falahi, Jalali & Babaee 2024; Gore et al. 2012). Characteristics such as age, sex, body mass index (BMI), physical inactivity (Kawabata et al. 2025) and general health have been reported to influence musculoskeletal disorders and exacerbate LBP symptoms. Evidence suggests that up to 82% of amputees experience LBP after amputation, underscoring the significant health burden associated with these musculoskeletal disorders (Sadowski et al. 2022). Compensatory gait strategies such as hip hiking or circumduction can further increase stress on lumbar structures, exacerbating the risk of biomechanical change (Highsmith et al. 2016; Wnuk-Scardaccione et al. 2023). Core muscle weakness also compromises postural stability, which further heightens susceptibility to LBP in this population (Matsumoto et al. 2019). In recent years, mobile health (mHealth) technologies have shown considerable promise in the management of musculoskeletal disorders (Quemeloa et al. 2023). Systematic reviews indicate that digital interventions can enhance physical function, with outcomes comparable to conventional face-to-face rehabilitation (Cottrell et al. 2017; Valentijn et al. 2022). Additionally, mHealth facilitates patient self-management, improves adherence to prescribed exercise programmes, and enables real-time interaction with healthcare providers. These advantages make mHealth a particularly relevant solution for populations with limited access to rehabilitation services (Frey et al. 2025; McSwan et al. 2021). Evidence also supports its benefits across a range of chronic musculoskeletal conditions (Slattery et al. 2019). Despite this potential, no mHealth application has been tailored specifically for lower-limb amputees, who present with unique rehabilitation needs. Current applications are typically generic, focusing primarily on education or monitoring, without addressing the distinct musculoskeletal disorders or integrating core-strengthening programmes essential for improving postural stability and quality of life in amputees (Agnew et al. 2022; Jang 2021; Lalloo et al. 2017; Wu et al. 2024). This gap underscores the need for targeted digital innovations that align with the rehabilitation requirements of this high-risk group. To address this limitation, the present study introduces mHealth, the first mHealth application specifically designed to support musculoskeletal health and enhance functional outcomes in individuals with lower-limb amputation. This intervention combines behavioural theory (Health Belief Model) with a hybrid model of community-based and remotely monitored core-strengthening exercises. By integrating education, structured physical training and social support, mHealth represents a novel approach to musculoskeletal rehabilitation and offers a new paradigm for applying mHealth to underserved populations with disabilities.

## Research methods and design

### Study setting

This study was conducted within the disability community organised under the Indonesian Association of Persons with Disabilities, Yogyakarta Branch, located in Sleman Regency, Yogyakarta. The community comprises individuals with various types of disabilities, including lower-limb amputation.

### Study participants

Participants were individuals with unilateral lower-limb amputation, either transtibial or transfemoral, who met specific eligibility criteria. Inclusion criteria were age 25–65 years, residence in Sleman Regency, ownership, and ability to operate a mobile phone, and willingness to participate until study completion by providing written informed consent. Exclusion criteria included severe comorbidities that could interfere with participation, communication difficulties, residence outside the study area or refusal to participate.

### Study design

A quasi-experimental one-group pre-test–post-test longitudinal design was conducted. All eligible participants were allocated to the intervention group. Descriptive assessments were performed at four time points: baseline, 1 month, 2 months and 3 months (end of follow-up), to summarise participant characteristics and to illustrate trends in outcomes over time using graphical displays. Because of the small sample size and non-normal distribution, nonparametric methods were applied. Exploratory bivariate analyses were conducted at baseline to describe relationships between selected variables and quality of life. Owing to the small sample size and non-normal distribution of the data, nonparametric methods were applied. Changes in quality of life between baseline and the 3-month follow-up were evaluated using the Wilcoxon signed-rank test. No regression-based or generalised estimating equation (GEE) analyses were performed.

### Study duration

The study was carried out over 3 months, from September to December 2024. The initial 2 weeks were allocated for recruitment and participant screening. This was followed by the 3-month intervention period, which included baseline data collection (pre-test), implementation of the mHealth application, biweekly evaluations and post-test assessment at the end of the intervention.

### Sampling strategy and sample size

A purposive sampling strategy was applied, selecting respondents according to the predefined inclusion and exclusion criteria. At the initial stage, 106 individuals with lower-limb amputation were screened. Eighty were excluded owing to refusal to participate, residence outside the study area, communication barriers (including difficulty using mobile applications) or severe comorbidities. A total of 25 participants met the criteria and received the intervention. During the study, three participants withdrew (two owing to work commitments and one owing to family issues), resulting in 22 participants included in the final analysis.

### Study procedure and intervention

**FIGURE 1:**
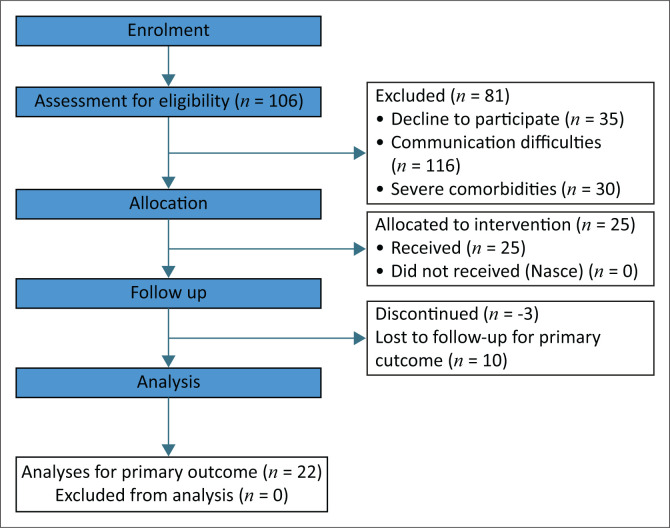
Consolidated standards of reporting trials (CONSORT) flow diagram.

Participants were recruited and screened according to eligibility criteria, after which informed consent was obtained and a baseline assessment was conducted, including measures of attitude, perceived susceptibility, perceived severity, core muscle strength and quality of life. The mHealth intervention consisted of integrated educational and exercise content delivered through a smartphone application over a 3-month period. The exercise component comprised instructional videos demonstrating 11 core-strengthening movements, each accompanied by 45 min of step-by-step explanations (three replications each set) and increased by 15 min in the following month to facilitate correct and safe performance at home. Participants were required to complete at least two exercise sessions per week, following the video guidance. The educational component provided practical information on safe body positions and movement strategies for daily activities (e.g. sitting, lifting and transfers) customised for lower-limb amputees. To support adherence, the application included automated reminders for scheduled exercise sessions and prompts to review the educational materials on a regular basis. All participants received the mHealth intervention and were evaluated biweekly for 3 months, assessing their adherence to the exercise regimen, specifically the number of exercise sessions performed, with a final assessment at the end of the study period to evaluate overall outcomes. The intervention was designed to improve core muscle strength and provide guidance on safe movement strategies. From 25 enrolled participants, three dropped out, resulting in 22 participants included in the final analysis (Figure 1).

### Intervention adherence and participant flow

The intervention was delivered over a 3-month period with two supervised sessions per week, resulting in a total of 24 scheduled sessions. All sessions were conducted under the supervision of trained instructors. Participant attendance was monitored using signed attendance sheets at each session and cross-checked with application usage records. A total of 25 participants were initially enrolled. Three participants attended fewer than four sessions (range: 1–3 sessions) and were considered withdrawn because they did not meet the minimum adherence criteria required for effective application use. The final analysis therefore included 22 participants who completed the intervention.

### Variables and instruments

Quality of life was assessed using the Indonesian version of the WHOQOL_BREF (translated by Catholic University Atma Jaya 2004; WHOQOL. 2012), which in this study comprised 26 items covering four domains: physical, psychological, social relationships and environment. The Indonesian version has been translated, culturally adapted and validated in previous studies. The Indonesian version of the WHOQOL-BREF has been widely used in previous Indonesian studies (Millenia et al. 2024; Resmiya & Misbach 2019) and has demonstrated good psychometric properties. Each item is rated on a five-point Likert scale. Domain scores were calculated according to the WHOQOL-BREF scoring guidelines and transformed to a 0–100 scale, with higher scores indicating better quality of life. In the primary analyses, quality of life scores were treated as continuous variables. For descriptive purposes, overall quality of life scores were also dichotomised into ‘high’ (≥ 60) and ‘low’ (< 60) categories based on previous studies (Silva et al. 2014; Silva et al. 2019). These categorical analyses were considered supplementary and exploratory. Baseline characteristics including age, duration of employment, education level, type of amputation and BMI were summarised descriptively.

### Data management and statistical analysis

Data were checked for completeness and consistency prior to entry, coded anonymously and verified using double entry. Data processing was performed with IBM SPSS Statistics version 29 (IBM Corp., Armonk, NY, USA). Given the quasi-experimental one-group pre-test–post-test longitudinal design, small sample size (*n* = 22) and non-normal distribution of outcome variables, nonparametric statistical methods were applied. Descriptive statistics and graphical displays were used to summarise participant characteristics and to illustrate trends in outcomes across four time points (baseline, month 1, month 2, and month 3). Pairwise changes between baseline and 3-month follow-up were analysed using Wilcoxon signed-rank tests. Bivariate associations between categorical predictors (attitude, perceived susceptibility, perceived severity and core muscle strength) and dichotomised quality of life were examined using chi-square or Fisher’s exact tests, as appropriate. Crude odds ratios (OR) and 95% confidence intervals (CI) were reported for descriptive and exploratory purposes only. Multivariable regression and GEE analyses were not performed because of the limited sample size and the high risk of overfitting. Adjusted analyses were considered statistically unreliable and were therefore avoided. Statistical significance was set at *p* < 0.05 (two-tailed).

### Ethical considerations

The study protocol, including the use of the mHealth application and procedures for collecting application (app) usage data, was approved on 29 July 2024 by the Health Research Ethics Committee of Hasanuddin University (protocol no.: 3724093039). Before enrolment, all eligible participants received verbal and written information about the study objectives, procedures, potential risks and benefits, and each participant provided written informed consent specifically for participation in the study and for the use of the mHealth intervention.

## Results

### Participant profile at baseline

A total of 22 participants were included in the study. The mean age was 46.3 ± 7.8 years (median 47; range 35–58 years). The majority were male (63.6%), and most had transtibial (below knee) amputation (68.2%). The mean duration since amputation was 6.8 ± 3.2 years (median 7; range 2–12 years). The mean BMI was 27.1 ± 3.9 kg/m^2^ (median 27; range 21–34), indicating a trend towards overweight. According to World Health Organization (WHO) categories, more than half of the participants were overweight (54.5%), followed by obese (22.7%), whilst only 22.7% had normal BMI. Furthermore, educational attainment varied, with the majority (63.6%) having completed at least senior high school. The causes of amputation were varied, such as amputee (sports-related injuries and occupational accidents) and congenital limb deficiency. The majority of all participants used a transtibial or transfemoral prosthesis in daily life and wore their prosthesis for at least 8 h per day on weekdays. This makes the study population well-suited to the aims of the mHealth application, which was designed to provide education and core-strengthening exercises. Baseline characteristics are summarised in Table 1.

**TABLE 1:** Baseline characteristics of participants (*N* = 22).

Variable	Total (*n* = 22)				Transtibial (*n* = 15)				Transfemoral (*n* = 7)			
	*n*	%	Years ± s.d.	kg/m^2^ ± s.d.	*n*	%	Years ± s.d.	kg/m^2^ ± s.d.	*n*	%	Years ± s.d.	kg/m^2^ ± s.d.
**Sex**												
Male	14	63.6	-	-	9	60.0	-	-	5	71.4	-	-
Female	8	36.4	-	-	6	40.0	-	-	2	28.6	-	-
**Education**												
< Senior high school	8	36.4	-	-	6	40.0	-	-	2	28.6	-	-
≥ Senior high school	14	63.6	-	-	9	60.0	-	-	5	71.4	-	-
**BMI categories**												
Normal (18.5–24.9)	5	22.7	-	-	3	20.0	-	-	2	28.6	-	-
Overweight (25–29.9)	12	54.5	-	-	9	60.0	-	-	3	42.8	-	-
Obese (≥ 30)	5	22.7	-	-	3	20.0	-	-	2	28.6	-	-
**Amputee device**												
Wheelchair	3	13.6	-	-	0	0.0	-	-	3	42.8	-	-
Prostheses	19	86.4	-	-	15	100.0	-	-	4	57.2	-	-
Congenital	4	18.1	-	-	4	26.6	-	-	0	0.0	-	-
Amputee	18	81.9	-	-	11	73.4	-	-	7	100.0	-	-
**Anthropometric**												
Duration since amputation	-	-	6.8 ± 3.2	-	-	-	6.5 ± 3.0†	-	-	-	7.2 ± 3.4‡	-
BMI	-	-	-	27.1 ± 3.9	-	-	-	26.8 ± 3.6†	-	-	-	27.7 ± 4.3‡
Age	-	-	46.3 ± 7.8	-	-	-	45.8 ± 8.1†	-	-	-	47.4 ± 7.3‡	-

BMI, body mass index; s.d., standard deviation.

† , Amputation level: Below knee;

‡ , Amputation level: Above knee.

Baseline characteristics of participants are presented using descriptive analysis. Continuous variables are summarised as mean ± standard deviation or median (range), as appropriate, whilst categorical variables are presented as frequencies and percentages.

### Intervention adherence and participant flow

Of the 25 participants initially enrolled, 22 (88.0%) completed the full intervention and were included in the final analysis. Three participants (12.0%) were excluded because of insufficient attendance (≤ 3 sessions). Participants who completed the programme attended all scheduled supervised sessions and consistently used the mHealth application as prescribed.

### Trajectory of outcomes across follow-up

**FIGURE 2:**
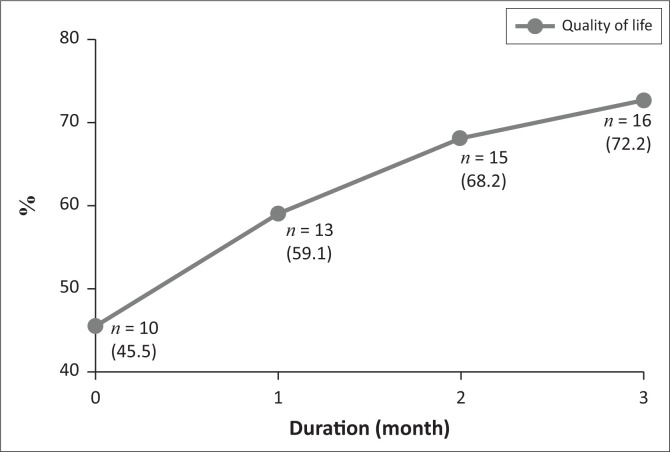
Descriptive trend in the proportion of participants with quality of life scores 60 across the follow-up time points.

Over the 3-month intervention period, both primary and secondary outcomes showed consistent within subject improvements. Descriptive analyses as shown in Figure 2 indicated significant overall changes across the four assessment points for quality of life, positive attitude and core muscle strength. The proportion of participants reporting good quality of life increased from 45.5% at baseline to 72.7% at month 3. Similarly, the proportion of participants with positive attitudes increased from 50.0% to 77.3%, and the proportion with good core muscle strength increased from 40.9% to 77.3%. Pairwise comparisons using Wilcoxon signed-rank tests showed significant improvements from baseline to month 3 in positive attitude and core muscle strength.

**FIGURE 3:**
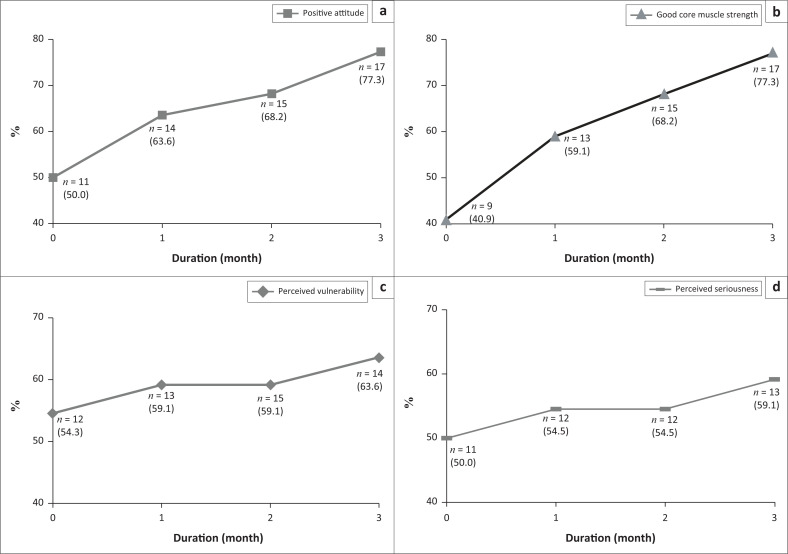
Descriptive trend in the proportion of participants with (a) positive attitude, (b) good core muscle strength, (c) perceived vulnerability, (d) perceived seriousness.

By contrast, perceived vulnerability and perceived seriousness did not demonstrate notable changes over the study period. This suggests that changes in quality of life occurred without corresponding changes in risk perception. Improvements in quality of life were observed concurrently with increases in positive attitudes and core muscle strength amongst lower-limb amputees using the mHealth application. Descriptive trends of these outcomes are presented in Figure 3.

### Bivariate associations between examined variables and quality of life

Exploratory bivariate analysis using the chi-square test (Table 2) indicated that positive attitude and core muscle strength were associated with quality of life at the baseline. The result showed that participants with positive attitudes showed higher odds of reporting good quality of life (OR = 2.9; 95% CI: 1.0–8.6; *p* = 0.045), and those with good core muscle strength also showed higher odds (OR = 4.5; 95% CI: 1.3–12.4; *p* = 0.018). In contrast, perceived susceptibility (OR = 1.8; 95% CI: 0.7–5.0; *p* = 0.162) and perceived severity (OR = 1.2; 95% CI: 0.5–3.5; *p* = 0.287) were not significantly associated with quality of life. These findings suggest that, at the bivariate level, behavioural (positive attitude) and physical (core muscle strength) factors were associated with quality of life at the bivariate level.

**TABLE 2:** Baseline bivariate associations between selected variables (*N* = 22).

Domain	Variable	OR	95% CI	*p*-value
Behavioural	Positive attitude	2.9	1.0–8.6	0.045
	High perceived vulnerability	1.8	0.7–5.0	0.162
Physical	High perceived seriousness	1.2	0.5–3.5	0.287
	Good core muscle strength	4.5	1.3–12.4	0.018

OR, odds ratio; CI, confidence interval.

### Wilcoxon signed-rank test

The Wilcoxon signed-rank test was used to evaluate within participant changes between baseline and 3-month follow-up. Wilcoxon signed-rank analysis (Table 3) revealed that participant change across variables behavioural and physical. In the behavioural variables, positive attitude showed a statistically significant improvement with a *W* = 15.0, *p* = 0.007 and a large effect size (*r* = 0.77), indicating improvement across participants. High perceived vulnerability demonstrated a low W value (*W* = 0.0) and a large effect size (*r* = 0.88) but did not reach statistical significance (*p* = 0.083). Moreover, in the physical domain, good core muscle strength improved significantly (*W* = 13.0, *p* = 0.020) with a large effect size (*r* = 0.79), indicating substantial improvement between baseline and follow-up. In contrast, high perceived seriousness showed no significant change (*W* = 10.5, *p* = 0.981) despite a large effect size (*r* = 0.80), suggesting variability in individual responses.

**TABLE 3:** Wilcoxon signed-rank test result (*N* = 22).

Domain	Variable	Wilcoxon	Effect size (*r*)	*p*-value
Behavioural	Positive attitude	15.0	0.77	0.007
	High perceived vulnerability	0.0	0.88	0.083
Physical	High perceived seriousness	10.5	0.80	0.981
	Good core muscle strength	13.0	0.79	0.020

## Discussion

This study suggests that the preliminary findings of mHealth intervention were associated with improvements in quality of life amongst lower-limb amputees over 3 months. Although this study did not measure LBP directly, improvements in core muscle strength and quality of life may be relevant to musculoskeletal health. The evidence showed in Wilcoxon signed-rank tests, which resulted in statistically significant improvements in positive attitude and good core muscle strength, both characterised by *p*-values < 0.05, and large effect sizes, indicating consistent and clinically relevant improvements across participants. Quality of life also improved significantly over time, supporting the relevance of these psychosocial and physical changes. These results also align with prior mHealth studies in musculoskeletal conditions, which reported enhanced quality of life through digital education (Zhang et al. 2025) and app-based exercises (Comachio et al. 2025). Practically, the application represents an innovative tool suitable for community-based rehabilitation programmes for individuals with disabilities. Positive attitude emerged as a significant factor, supporting the Health Belief Model framework, where attitudes influence health behaviour adoption. Educational modules in the application were crucial in shaping positive attitudes and promoting active participation (Mota et al. 2025; Zhou, Salman & McGregor 2024), consistent with prior studies highlighting psychosocial determinants of adherence to musculoskeletal interventions (Zhou et al. 2024). Moreover, a study performed by Quemelo et al. (2023) showed that mHealth education and exercise can improve musculoskeletal health, function and quality of life. The increase in QoL may be attributed to improvements in core muscle strength and postural stability, which support functional mobility and overall musculoskeletal health LBP in amputees can result from internal biomechanical factors, such as muscle strength, gait and postural alignment. This is because amputees rely heavily on their remaining muscles, particularly in the core and upper body, to compensate for the missing limb. This reliance often leads to compensatory gait patterns and abnormal postural positions, which can place additional strain on the lumbar spine, causing spinal misalignment and muscle fatigue. This may explain the increase in core muscle strength after exercise treatment, which is associated with better quality of life (Kheirinejad et al. 2023). Integrated core exercises enhanced postural stability and reduced spinal load (Hlaing et al. 2021; Li et al. 2025). These findings are consistent with literature showing core strengthening as effective in musculoskeletal rehabilitation and amputee populations (Cheng et al. 2025; Shin et al. 2018). However, perceived susceptibility and severity were not significantly associated with quality of life, suggesting that awareness alone does not drive meaningful change unless it is paired with behavioural and physical interventions. This aligns with studies showing that perception does not always translate into behaviour change. Accordingly, the mHealth application is most effective when it combines interactive education with structured exercises (Ju et al. 2022). Mobile health applications encourage self-management by providing patients with tools and resources necessary to actively manage their condition. A systematic scoping review indicates that mHealth enables individuals to engage in self-care, enhancing their understanding and motivation to adopt healthier lifestyles, which are crucial for effective musculoskeletal health management (Rintala et al. 2022). These applications often include personalised exercise programmes, pain diaries and educational content designed to increase patient engagement and adherence to therapeutic regimens (Hernandez-Lucas et al. 2023). Mobile health showed promising evidence education and exercise routines integrated into daily life to improve musculoskeletal conditions (Andargeery & El-Rafey 2025; Sañudo et al. 2025) and beneficial for individuals with LBP (Ulrich et al. 2023). Despite the study showed promising results, it is crucial to consider potential confounding factors and threats to internal validity when interpreting these findings. Critical factors such as baseline physical activity levels (Heneweer et al. 2011), comorbidities (Gandløse et al. 2025), detailed patterns of prosthetic use (Livdans-Forret 2005), socioeconomic status and prior occurrences of LBP were not fully assessed. For individuals with amputations, especially those using prosthetics, the design of the prosthesis can also influence LBP. The biomechanical efficiency of a prosthesis can promote or hinder proper gait behaviour (Jayaraman et al. 2018). It also can lead to modified gait mechanics, potentially imposing additional stress on the lumbar spine and surrounding muscles (Nolasco, Silverman & Gates 2024). Furthermore, the changed walking patterns of participants with prosthetics engaged with biomechanical impact and could contribute to LBP, especially if prosthetic alignment or fit was suboptimal. The mechanism of incorrect posture and gait deviations causes overcompensation in certain body areas, particularly the lower back. These factors may have influenced both engagement with the mHealth intervention and the observed quality of life outcomes. Moreover, the quasi-experimental one-group pre-test – post-test longitudinal design is susceptible to various threats, including historical effects (e.g. concurrent changes in participants’ lives or access to care), natural maturation or adaptation over time and the Hawthorne effect, wherein participants may modify their behaviour solely in response to observation and support from the research team.

### Limitations

This study has several important limitations. Firstly, the small sample size limited statistical power and generalisability. Secondly, dichotomising continuous WHOQOL-BREF scores into ‘good’ versus ‘poor’ quality of life likely reduced statistical power and may have masked more nuanced changes in quality of life. Thirdly, a quasi-experimental one-group pre-test–post-test longitudinal design lacking a control group precludes causal inference, and any observed benefits over time cannot be unequivocally ascribed to the mHealth intervention and may be partially as a result of natural adaptation or other unmeasured factors. Fourthly, although adherence was monitored using attendance records, detailed objective app usage metrics were not available. Several potentially relevant factors, including prosthesis type and prosthetic alignment or fit, were not measured in this study. These factors may influence functional outcomes and quality of life and should be considered in future studies.

## Conclusion

The findings provide preliminary evidence that a tailored mHealth intervention can be associated with favourable changes in behavioural and physical determinants of quality of life in lower-limb amputees. Compared with other digital interventions, such as virtual reality-based pain education, this approach shows that digital health technologies can be tailored to specialised populations with promising outcomes. Strengths of this study include the innovative design of a dedicated mHealth application, longitudinal repeated-measures design and use of statistical analysis.
